# Effect of Hydro-alcoholic Extract of Persian Oak (Quercus brantii) in Experimentally Gastric Ulcer 

**Published:** 2014

**Authors:** Shahrzad Azizi, Abdollah Ghasemi Pirbalouti, Mahdi Amirmohammadi

**Affiliations:** a *Department of Pathobiology, Veterinary Medicine Faculty, Shahid Bahonar University of Kerman, Kermn, Iran. *; b *Department of Medicinal Plants, Shahrekord Branch, Islamic Azad University, Shahrekord, Iran. *; c *Medicinal Plants Program, Collage of Natural Science, University of Massachusetts, Amherst, MA, 1003, USA. *; d *Young Research Club, Islamic Azad University, Jiroft Branch, Kerman, Iran. *

**Keywords:** *Quercus brantii *Lindl, Histopathology, Gastric ulcer, Rat

## Abstract

Persian oak (*Quercus brantii *Lindl.) belongs the family Fagaceae, is a medicinal plant which seed flour is used to treat inflammatory and gastric ulcers by the tribes in south western Iran. The current study was done to evaluate the effect of hydro-alcoholic extract of *Q. brantii *seed flour for treatment of gastric ulcers induced by ethanol in Wistar rats. The hydro-alcoholic extract of *Q. brantii *was tested orally at doses of 250, 500, and 1000 mg/Kg, control group and standard drug (omperazole) on experimentally gastric ulceration. At the 3, 6, 9, and 14^th ^days, ulcer index in mm^2^ and histopathological findings were evaluated. Results indicated the size of ulcers significantly reduced at 9, and 14 days after of *Q. brantii *extract treatment. Curative effect in the hydro-alcoholic induced gastric damage was 100% at 1000 mg/Kg and omeprazole, 99.8 % at 500 mg/Kg, and 95.4% at 250 mg/Kg after 14 days. Results of histopathological investigation showed the thickness of ulcerated mucosa was similar to the normal mucosa with 1000 mg/Kg of *Q. brantii *hydro-alcoholic extract after 14 days but in the groups treated by 250, and 500 mg/Kg, superficial erosions were visible in the central portion of the healed ulcers. In conclusion, the hydro-alcoholic extract of *Q. brantii *had active components (tannin = 8.2%) that accelerates ulcer healing and thus supported its traditional use.

## Introduction

Gastric ulcer is a common disorder of digestive system that a considerable number of people in the world are affects. Current chemical drugs using for treatment of gastric ulcer are associated with many side effects. Recently, developing of new drugs with herbal origin is considered for decline of chemical drugs side effects (1). In folk knowledge, medicinal plants are used for treatment of various disorders with little information of their pharmacological uses (2). 

*Quercus *is the most frequent genus of the Fagaceae family in forests of Iran (3). Several species of oaks grow abundantly in Zagros, Arasbaran and Hyrcanian forests exhibiting remarkable morphological variation. The main species of Chaharmahal va Bakhtiari forests is *Q. brantii*, which is widely distributed all over the area, particularly between 700 and 2400 m of altitude above sea level. *Q. brantii *popularly known as Persian oak in English and “Balout Irani” in Persian used for treatments gastric disorders by traditional people in Zagros region (Chaharmahal va Bakhtiari, Ilam and Kohghiluyeh va Boyer Ahmad provinces), southwestern Iran (4, 5, 6). The seed hulls of the Persian oak have been used as an anti-diarrheic remedy in traditional medicine. The tannins extracted from *Q. brantii *have contractive and disinfectant effects (7). The extracts isolated from *Q. brantii *seed and seed hulls have been shown to have biological and pharmacological activities such as anti–bacterial (8, 9, 10), wound healing effect (11). 

To our knowledge, no documented reports on the hydro-alcoholic extract of *Q. brantii *seed on gastric ulcers are available. Therefore, the aim of this study was to evaluate the effect of hydro-alcoholic extract of *Q. brantii *seed flour for treatment of gastric ulcers induced by ethanol in Wistar rats.

## Experimental

*Collection and identification of plant material*

Fruits of Persian oak (*Quercus brantii *Lindl., the Fagaceae family) were collected in September 2011 from Ardal region in central Zagros mountains (latitude. 31° 50´, longitude. 51° 23´, altitude. 2120 m above sea level), Chaharmahal va Bakhtiari province, Iran. Plant identities were confirmed by Dr. H.A. Shirmardi, and a representative voucher specimen (IAUSHK-351) was been placed in the Herbarium of Research Center of Medicinal Plants and Ethno-veterinary, I.A.U, Iran. 

*Plant extract preparation*

Seeds of Persian oak were dried inside for one week at room temperature (30 ± 5 °C), and the ground to fine a powder using Moulinex food processor. The hydro-alcoholic extract was obtained by maceration of the crude plant powder with ethanol/water (70/30) for four days in a chamber temperature (35 ± 5 °C) in the dark. The extract was filtered using a sterile cloth sheet. The filtrate was evaporated under reduced pressure at temperature below 45 °C with a rotary evaporator and a dark green hydro-alcohol extract (yield: 14.1%) was obtained. Phytochemical screening of the extract revealed the presence of tannins. The extract samples were stored in universal bottles and kept at 4 °C prior to use.

*Ethanol induced ulcer*

Male Wister rats (200-250 g) of three months were used. The animals were housed in standard environmental conditions of temperature (22 ± 3 ºC), humidity (60 ± 5%) and a 12 h light/dark cycle. During experimental time rats were given standard pellet diet (Pastor Institute, Iran) and water *ad libitum*. The rats were used for the experiment after one week of acclimatization period. All the procedures were approved by the Medical Ethics Committee of Shahrekord University of Medical Sciences. The rats fasted for 48 h and supplied with sucrose 8% during the fasting period. Each animal received 1 mL of absolute ethanol (99.6%) orally (12, 13). The animal divided in groups including *Q. brantii *extracts (250, 500, and 1000 mg/Kg), omeprazole (20 mg/Kg), and distilled water (control negative). Treatment was performed orally after 1 h of ulcer induction until 14 days.

*Determination of ulcer index*

On 3, 6, 9, and 14 days after induced ulcer, experimental rats were euthanized and then necropsied. After excision of the abdomen, the stomach was opened along the greater curvature and washed by normal saline. Ulcer areas on the glandular and non-glandular of gastric mucosa were examined macroscopically. Graded transparency sheet with square millimeter paper was put on stomachs› surface and investigated under a dissecting microscope. The ulcerous areas were drawn on sheet. The sum of the ulcerous areas was expressed in mm2 as the ulcer score. Curative effects of the *Q. brantii *extracts were evaluated with the following formula and compared with the results obtained from control and omeprazole groups (14).

$\mathrm{Curative ratio}=\frac{\left(\mathrm{Control ulcer index}\right)-(\mathrm{test ulcer index})}{\left(\mathrm{Control ulcer index}\right)}\times 100$

*Pathologic investigation*

At the 3, 6, 9 and 14^th^ days, experiment was terminated, and the ulcer area was removed from the surviving animals for histological examination. Tissue samples from each suspected lung and regional lymph node (1 × 1 x 0.5 cm^3^) were fixed in 10% neutral buffered formalin for histopathological examination. The samples were then dehydrated in graded ethanol and embedded in paraffin. Sections of 5 μm in thickness were stained with hematoxylin and eosin and examined by an ordinary light microscope (Olympus BX51).

*Statistical analysis*

The results were expressed as mean ± SD. The differences between ulcer indexes of experimental groups were rested by using ANOVA. Means of ulcer index were compared by Tukey-test at p ≤ 0.05 level. All data processing was performed with SPSS software (version 11.5).

## Results

*Ulcer index*

The analysis variance indicated significant differences (*p *≤ 0.01) among different experimental and control groups for ulcer index on 3, 6, 9 and 14 days after ulcer induction (Table 1). At 3^th^ day, ulcer index score decreased significantly from 321.7 ± 17.9 mm^2^ in negative control to 128.3 ± 9.27, 90.0 ± 12.16, 186.66 ± 43.71, and 150.00 ± 33.20 mm^2^ in animals treated with the hydro-alcoholic extracts of *Q. brantii *at 250, 500, and 1000 mg/Kg and standard drug (omeprazole). At 6th day, ulcer index score decreased significantly from 251+26.8 mm2 in negative control to 79.33 ± 24.67, 64.66 ± 7.42, 73.00 ± 18.00, and 51.00 ± 11.84 mm^2^ in animals treated with the hydro-alcoholic extracts of *Q. brantii *at 250, 500 and 1000 mg/Kg and standard drug. At 9^th^ day, ulcer index score decreased significantly from 285.66 ± 7.85 mm^2^ in negative control to 59.33 ± 8.29, 17.33 ± 6.35, 10.33 ± 4.91, and 19.66 ± 8.19 mm^2^ in animals treated with the hydro-alcoholic extracts of *Q. brantii *at 250, 500, and 1000 mg/Kg and standard drug. At 14^th^ day, ulcer index score decreased significantly from 314 ± 17.75 mm^2^ in negative control to 14.33 ± 2.96, 0.33 ± 0.33, 0.00, and 0.00 mm in animals treated with the hydro-alcoholic extracts of *Q. brantii *at 250, 500, and 1000 mg/Kg and standard drugs. Generally, the results indicated that omeprazole, the hydro-alcoholic extracts of *Q. brantii *at 1000, 500, and 250 mg/Kg had 100%, 100%, 99.8%, and 95.4% healing effect after 14 days induced ulcer, respectively.

**Table 1:** Effect of *Quercus brantii *extract on ethanolic gastric ulcer in rats

***p***	**Curative Ratio (%)**	**Ulcer index (mm** **2** **)**	**Dose (mg/Kg)**	**Test Samples**	**No of Animals**	**Treatment duration **
0.05<	- 53.3 60.1 72.2 41.9	321.66 ± 17.90 ^b^* 150.00 ± 33.20 ^ab^ 128.33 ± 9.27 ^a^ 90.00 ± 12.16 ^a^ 186.66 ± 43.71 ^ab^	- 20 250 500 1000	Ethanol + D.W Ethanol + Om Ethanol + QB Ethanol + QB Ethanol + QB	3 3 3 3 3	3 days
0.01<	- 79.6 68.4 74.2 70.9	251.0 ± 26.83 ^b^ 51.00 ± 11.84 ^a^ 79.33 ± 24.67 ^a^ 64.66 ± 7.42 ^a^ 73.00 ± 18.00 ^a^	- 20 250 500 1000	Ethanol + D.W Ethanol + Om Ethanol + QB Ethanol + QB Ethanol + QB	3 3 3 3 3	6 days
0.01 <	- 93.1 79.2 93.9 96.3	285.66 ± 7.85 ^c^ 19.66 ± 8.19 ^a^ 59.33 ± 8.29 ^b^ 17.33 ± 6.35 ^a^ 10.33 ± 4.91^a^	_ 20 250 500 1000	Ethanol + D.W Ethanol +Om Ethanol + QB Ethanol + QB Ethanol + QB	3 3 3 3 3	9 days
0.01<	- 100 99.8 95.4 100	314.66 ± 17.75 ^b^ 0 ^a^ 14.33 ± 2.96 ^a^ 0.33±0.33 ^a^ 0 ^a^	_ 20 250 500 1000	Ethanol + D.W Ethanol + Om Ethanol + QB Ethanol + QB Ethanol + QB	3 3 3 3 3	14 days

Note: results are mean±SD.

* Similar letters show no significant difference between groups.

*Evaluation of histopathologic findings*

At 3^th^ day of treatment, all experimental and control groups indicated severe mucosal ulceration, necrosis and hemorrhage grossly. Ulcers in different forms and sizes were observed principally in glandular and lesser in non-glandular part of stomach. The mucosal stomach showed hyperemia. The margins ulcers were swollen due to edematous. Hyperemia was more evident in the control groups than in the others. Histopathologically, gastric mucosa was sloughed due to necrosis, and erosions and ulcers in different degrees were occurred. Lamina properia showed hyperemia and hemorrhage. Around the marginal ulcers, inflammatory cells especially neutrophils and a few eosinophils infiltrated. Submucosa layer was edematous and thickened by aggregation a lot of fibrinohemorrhgic exudates.

At 6^th^ day, the hyperemia and hemorrhage were decreased in all treated groups especially the group treated by omeprazole. A fibrinonecrotic layer was covered various sizes of ulcers and erosions superficially. The control group showed severe hemorrhage and hyperemia with severe ulcers. At the base of ulcerated areas, granulation tissue developed. Lamina properia and submucosa layer were moderately hyperemic and inflammatory cells were present in these layers.

At 9^th^ day, mucosal hemorrhage and hyperemia were decreased remarkable in the hydro-alcoholic extracts of *Q. brantii *at 500, and 1000 mg/Kg and omeprazole group in comparison to control group. The ulcerated lesions were repaired but small erosions were remained yet. The treated group by 250 mg/Kg extract of *Q. brantii*, indicated more hemorrhage and hyperemia, and presence of some ulcers were noticeable. Thickness of gastric mucosa was variable in different areas. Microscopic study indicated proper regeneration of epithelium, especially in the edges of ulcers. New formed gastric glands had simple columnar epithelium with dilated lumens. In the central portion of the healed ulcers superficial erosions were visible. A dense connective tissue scar was observed at the base of healed ulcer. In the submucosa layer, a great number of newly formed vessels were considerable in the extracts treated groups, especially at the higher doses.

**Figure 1:**
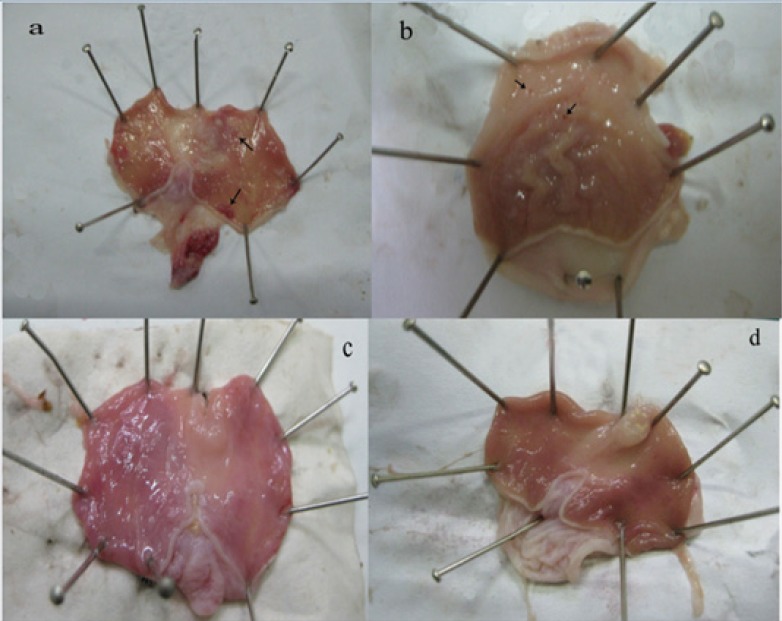
Ethanol-induced ulcers in rat’s stomach 14 days after treatment; a-d) rat treated with *Quercus brantii *extract 250, 500, 1000 mg/Kg and omeprazole (20 mg/Kg) respectively. Macroscopic small erosions and ulcers are visiblein treated rat with 250 and 500 mg/Kg extract (arrows).

**Figure 2:**
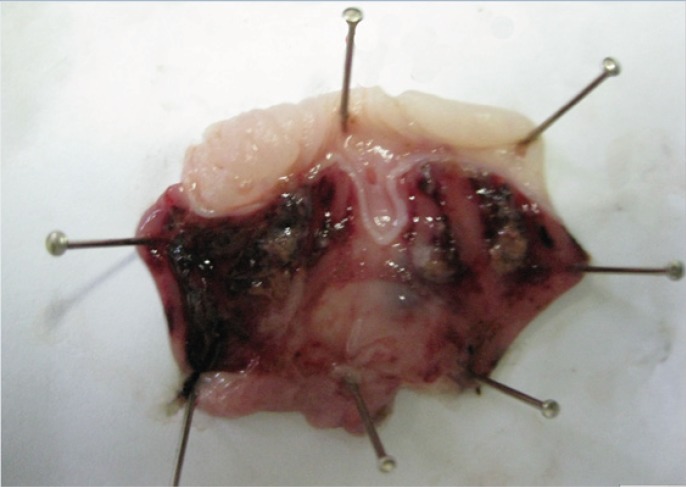
Severe gastric erosions, ulcers and hemorrhage induced by ethanol in ratafter14 days (negative control group treated by saline).

**Figure 3:**
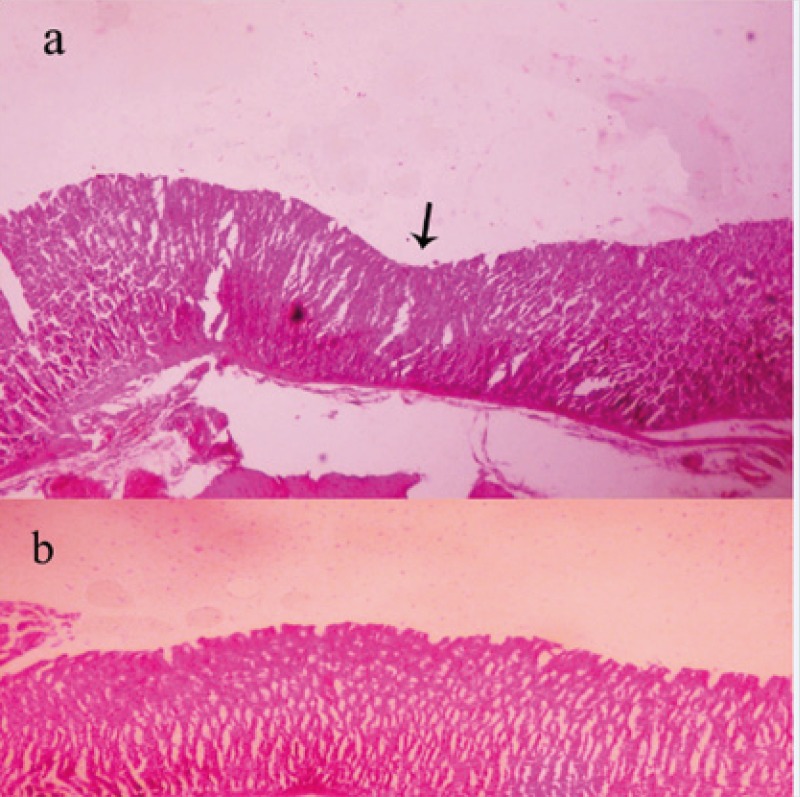
Histopathologic features of stomach in rat treated with *Quercus brantii *extract (250 and 500 mg/Kg) show remained superficial erosion in the central part of healed ulcer (arrow) in comparing with dose 1000 mg/Kg of extract (Figure b) (×100, H-E).

At 14^th^ day, no gastric erosions or ulcers were found in the rats which received the extract of *Q. brantii *at 1000 mg/Kg, and omeprazole but very small erosions were visible yet at dose 250 and 500 mg/Kg of extracts (Figure 1). Control group showed sever hemorrhage and mucosal lesions (Figure 2). We observed a thicker regenerative mucosa in the treated animals with 1000 mg/Kg and omeprazole in comparing to the groups treated with 250 and 500 mg/Kg for 14 days. Very small superficial erosions were observable yet in the central portion of the healed ulcers (Figure 3). In all groups, there were infiltration of a few lymphocytes and eosinophils in the submucosa layer and the base of repaired areas.

## Discussion

In ethno-pharmacology knowledge, study on natural herbal products is aimed for improvement of new drugs because chemical drugs are expensive and have several disadvantages. Attention is focused on components with less price and toxicity that may be more effective. Medical plants are a candidate for substitution with currents drugs (15). In folk medicine, some *Quercus *species is a medical plant with various therapeutic properties specially treatment of gastritis (16-18). The current study investigated the healing effect of *Q. brantii *on gastric ulcers induced by ethanol in rats. In experimental studies, intra-gastrically ethanol uses as the most common ulcerogenic agent that lead to severe erosions and ulcers in the stomach (19). The pathogenesis of ethanol-induced gastric ulcers is multifactorial. It causes mucus content of gastric wall is depleted (20). Ethanol disrupts the mucosal barrier of gastric wall with induction of damage to microvessels (21, 22). In addition, some agents such as indomethacin, alcohol, and aspirin produce oxygen free radicals and enhance lipid peroxidation (23). A powerful relationship has been observed between lipid peroxidation and ulcer formation in the stomach (24). Ethanol induces gastric damages through the oxidative stress and producing of reactive oxygen species (25). Results of our study indicated the hydro-alcoholic extract of *Q. brantii *extract decreased index ulcers significantly at all doses (250, 500, and 1000 mg/Kg). The macroscopic measures of ulcers showed that the *Q. brantii *extract repaired ethanol induced gastric lesions dose dependently. These results indicate a relationship between extract dosages and time. Microscopically, the thickness of ulcerated mucosa was similar to the normal mucosa with 1000 mg/Kg of *Q. brantii *extract after 14 days but in 250, and 500 mg/Kg dose of extract very small superficial erosions were observed in the gastric wall after 14 days of treatment. Several studies (26, 27, 28, 29, 30) on the gastro-protective effect of crude drugs extracts and herbal mixtures suggest that most studies can be undertaken with doses in the range 100–800 mg/Kg of both crude drug extracts and herbal mixtures. Even at those doses the extract amount administered is a multiple of the traditional doses. In agreement with our study, review literature shows different species of *Quercus *have therapeutic effects on gastric ulcer. Ebrahimi *et al. *(2012) investigated the antibacterial and wound healing effects of methanolic extract of *Q. persica *fruits in three concentrations (25, 50, and 75 mg/mL) in rats. Their results showed that all concentrations were effective on the inhibition of *Staphylococcus aureus*, *S. epidermidis, *and *Escherichia coli *but the effect of 50 and 75 mg/mL extracts was significant for bacteria. In addition, the treated wounds with extract indicated better epithelialization, and wound contraction in comparison to control wounds. They suggested *Q. persica *possesses compounds with antibacterial and wound healing properties (11). Khouzami *et al*. (2009) investigated gastro-protective effect of *Q. infectoria *extract bark for two days. They reported (56–67%) protection against induced gastric lesions by ethanol (16). Gastro-protective and curative effects of *Quercus *spp. against ethanol-induced gastric damage were also attributed to other species of *Quercus *including extract from *Quercus ilex *root bark (31, 32), *Q. coccifera *and, *Q. aegilops *fruits (17). Khennouf *et al. *(2010) studied the anti-lipoperoxidant activity of some phenolic acids, flavonoids and purified tannins from *Quercus *spp. They described the phenolic acids, flavonoids and purified tannins exhibit gastro-protective properties by acting as inhibitors of lipid peroxidation process (33). These materials showed anti-lipoperoxidant activity. This antioxidant property is believed to be responsible for the gastro-protective effects of *Quercus *tannins and phenolic compounds. In addition, reported the polyphenols such as tannic acid, quercetin and ellagic acid in *Q. infectoria *bark, are able to inhibit the proton pump present in paretial cells and resulting in decreasing of gastric acid secretion (18, 31, 32). Khennouf *et al*. (2003) used extracted tannins from leaves of *Q. suber *and *Q. coccifera *orally in mice and observed this component prevented the gastric lesions induced by ethanol (18). In current study, the presence of tannin 8.2% based dry weight in seed flour of *Q. brantii *may be responsible for the curative action of the hydro-alcoholic extract of *Q. brantii *seed. The animals treated with the fruit extract at a higher dose of 1000 mg/Kg did not manifest any significant abnormal signs and changes in behaviors. They had no mortality and decreasing of weight during the course of the experiment. Further studies are needs for detection of different components of *Q. brantii *extract and demonstration of their therapeutic effects of each purified substance. 

In conclusion, we can suggest the hydro-alcoholic extract of *Q. brantii *seed is effective in experimentally healing rat ulcers. The hydro-alcoholic extract of *Q. brantii *seed at 250, and 500 mg/Kg produced a significant gastric ulcer when compared with the negative control. We suggest the extract of *Q. brantii *seed and more appropriately the active compounds should be assessed for action mechanisms to elucidate their mode of action. Furthermore, new action mechanisms may be discovered. Hence, the results of current study support the traditional uses of *Q. brantii *to treat gastric ulcer. 
